# 

**Published:** 1894-04

**Authors:** 


Examinations U. S. Army Medical Corps.—In view of the possibility of the reduction of the Medical Corps from 125 to ninety Assistant Surgeons, by action of Congress at its present session, and to save possible loss of time and expense to candidates if such action be taken, the examinations appointed for March and April, 1894, will, by order of the Secretary of War, not be held until further notice.

It is probable that if the Corps should not be reduced, the Examining Board will be convened in the fall of 1894. Of this, notice as early as possible will be given.

---

THE whole world has been traversed to find material for the Easter Number of *The Literary Digest.* Almost every civilized language is represented. It is superbly illustrated, full of information; treating all questions of present interest, and all sides of those questions; presenting the leading articles in the foremost magazines and journals of the world. This number of *The Literary Digest* probably excels any other attempt to give the literature of the world in one issue. The Easter number was ready on Thursday, March 22d.

---

WE HAVE received one of Hewson's Anomaly Blanks, designed for the use of dissectors, demonstrators of anatomy or post-mortem examiners in which to record any anatomical anomaly that they may find. The sheet is nine by six inches, has proper spaces for the dissector's name, the principal normal physical characteristics of the subject and for anomalies of each of the principal organs or classes of tissues. These blanks are supplied in blocks by the publishers, Messrs. P. Blakiston, Son & Co., Philadelphia, free to any professor or demonstrator of anatomy in any medical college.

---

DR. CHARLES N. SMITH, editor of the *American Gynecological Journal,* published at Toledo, Ohio, announces the discontinuance of that magazine. We regret to part with this useful periodical, but the increasing professional duties of Dr. Smith interfered with the exacting demands that the journal made upon his time.

---

THE Angier Chemical Co., of Boston, is sending to the medical profession a card of excellent pens made by the Tadella Pen Co., 76 Fifth avenue, New York, which the company will take pleasure in forwarding on application to any physician who has not already received one.

THE *International Medical Magazine* has been transferred by J. B. Lippincott Co. to the International Medical Magazine Co., publishers, 716 Filbert street, Philadelphia. It is edited under the supervision of Drs. John Ashurst, Jr., and James T. Whittaker by Henry W. Cattell, M. D. Manuscripts, exchanges and books for review should be addressed to the editorial office, 3455 Woodland avenue, Philadelphia.

---

WE INVITE attention to the advertisement of the Labordine Chemical Co., that appears for the first time in this journal and will be found on our third cover page. We hope this new vegetable preparation will receive thorough investigation as to its merits at the hands of our physicians. If a satisfactory and safe substitute can be found for the numerous coal-tar preparations it will prove most desirable.

---

THE Woman's Medical College of Baltimore publishes its announcement on page xvi. of our advertising columns. There is a constant increase in medical colleges for women which indicates a demand for them. We hope that the standard provided by these colleges both for admission and graduation will not be made lower than in colleges for men.

---

THE *Louisville Medical Monthly* is the title of a new medical journal, published in Louisville, Ky., that appeared in March, 1894. Its editors are Drs. James B. Steedman and George M. Warner. In its list of active collaborators are included the names of some of the most prominent physicians of Louisville.

---

A WORK that is really new, and unlike any other, is *The Nutshell Cyclopedia*. It is not intended to take the place of any other general cyclopedia, but to *supplement every other*, and whatever other you have you will probably want the *Nutshell*, and if you have no other you will surely want it. It treats only *live* subjects, the facts concerning which are constantly changing, and the *latest* information concerning which is important. Probably three-fourths of its contents will be found in no other cyclopedia, because the occurrences and the facts described are of later date than the publication of any other cyclopedia. Though the entire work will comprise probably 2,500 pages, it is sold at a price so low that anyone can afford it, ranging from $1.75 for the complete work in

Monthly Parts, to $3.20 for the same in four volumes, half morocco binding. The issue before us covers such important subjects as coinage, Colorado, Columbia celebrations and expositions, commerce and crops, bringing statistics down to 1894 (mostly a year later than to be found in any of the annuals), Congress, with list of the members, Connecticut, and so on. For sample Monthly Part send 4 cents postage to John B. Alden, publisher, 57 Rose street, New York.

---

THE SHADOW-TEST.—A course of lectures, demonstrations and clinical work on skiascopy, or the shadow-test, will be given at the Philadelphia Polyclinic during the week commencing April 9th. This method of determining the refraction of the eye has for years been practised as a part of the regular routine examination in that institution; and is there found to be of greater practical value than the methods by the use of the ophthalmoscope or the ophthalmometer.

---

VICK'S FLORAL GUIDE, 1894.—It contains descriptions that describe, not mislead; illustrations that instruct, not exaggerate. This year it comes to us in a suit of gold. Printed in eight different colors besides black. Colored plates of chrysanthemums, poppies and vegetables. On the front cover is a very exquisite bunch of Vick's new white branching astor, and on the back is the new double anemone; 112 pages filled with many new novelties of value as well as all the old leading varieties of flowers and vegetables.

We advise our friend who intends doing anything in the garden this year to consult Vick's before starting operations. Send 10 cents to James Vick's Sons, Rochester, N. Y., for Vick's Guide; it costs you nothing, as you can deduct the 10 cents from first order. It certainly will pay you.

---

EDWARD BOK'S successful article in the January *Cosmopolitan* on The Young Man in Business has been reprinted in a tasteful and handy booklet form at ten cents by The Curtis Publishing Company, of Philadelphia. To this reprint Mr. Bok has added some fourteen pages of editorial matter answering Three Uncertain Young Men.

